# Comparison between the English and Bahasa Malaysia language versions of the Visual Functioning Questionnaire (VFQ-25) for use in patients with cataracts

**DOI:** 10.1186/s12886-021-02100-4

**Published:** 2021-09-27

**Authors:** Thanigasalam Thevi, Adinegara Lutfi Abas, Chang Stephanie Yen Li

**Affiliations:** 1 Hospital Melaka, Jalan Mufti Haji Khalil, 75400 Melaka, Malaysia; 2 MAHSA University, Jalan SP2, Bandar Saujana Putra, 42610 Selangor, Malaysia; 3 Manipal University College Malaysia, Jalan Padang Jambu, 75400 Melaka, Malaysia

**Keywords:** trends- cataract, visual functioning questionnaire, factor analysis

## Abstract

**Background:**

We conducted the study to compare the psychometric properties of the English version of the Questionnaire and the Bahasa Malaysia (Malay Language) version regarding the vision-related Quality of Life of patients with cataracts.

**Methods:**

The Malay version was translated by two independent translators who were well versed in both languages. We carried out a cross-sectional study collecting data between June 2017 and March 2018 in the pre-operative Eye Clinic of Hospital Melaka with 224 respondents (mean age 66.8 years) and another 204 respondents (mean age 64.3 years) participating in the English version and Malay version of the Questionnaire respectively.

Methods used to validate the standard questionnaire included the use of construct validity via factor analysis and the deployment of reliability test through assessment of internal consistency via Cronbach’s alpha.

**Results:**

We observed both English and Bahasa Malaysia versions to have high reliability with Cronbach’s alpha values of 0.90 and above in factors on difficulty with activities and responses to vision problems.

Exploratory factor analysis performed revealed that the three-factor model fits the data well for the English version of the questionnaire - difficulty with activities (23.81 % of variance), responses to vision problem (22.22 % of variance) and general health and vision (14.68 % of variance).

The Bahasa Malaysia version of the questionnaire produced three factors with two of the factors resembling the factors from the original version of the questionnaire - difficulty with activities (24.3 % of variance) and responses to vision problem (23.7 % of variance).

Item response theory analysis revealed that these factors for both English and Bahasa Malaysia versions comprised of adequately fitted items.

**Conclusion:**

The present study observed that both the English and Bahasa Malaysia versions of the NEI VFQ-25 have comparable construct validity to the original American version. With high validity and reliability, the tool shall be able to provide health care providers the assessment of impact due to cataract and other ophthalmic conditions on the vision-related quality of life of ophthalmic patients.

## Background

Cataract which is defined here as opacity of the lens has been noted as the leading cause of blindness contributing 39 % of total blindness in Malaysia [1]. The optimal treatment for cataract is cataract surgery. Challenges towards the implementation of surgical services in resource-limited environments are substantial and include limited human resources, transportation systems, and access to electricity and water [2]. Cost-effectiveness plays a role in establishing and carrying out surgical services [3].

Monocular impairment and better-eye acuity were associated with a decrease in most domains representing quality of life [4]. A substantial increase in falls and fall injuries and poorer health related quality of life were found in the elderly with cataracts while waiting for surgery [5]. A significant difference was observed between having cardiovascular diseases, respiratory and gastrointestinal diseases, hearing and visual impairments with poorer total score in the quality of life [6].

There have been various questionnaires developed to assess psychometric properties of illnesses that causes deterioration of vision. Patients with diseases such as diabetic retinopathy, cataract, primary open angle glaucoma, optic neuritis and uveitis have been included in research that evaluate vision related quality of life utilizing questionnaires that were deemed to be valid and reliable such as the VFQ-25 (questionnaire with 25 questions) and VFQ-51 (questionnaires with 51 questions) [7–9].

We aimed to compare the psychometric properties of the English version of the National Eye Institute Visual Functioning Questionnaire − 25 (VFQ-25) and the Bahasa Malaysia (Malay Language) version. In this study, the National Eye Institute Visual Functioning Questionnaire − 25 (VFQ-25) is primarily used to look into assessment of problems of vision and perception of quality of life among patients with cataracts.

## Methods

We carried out a cross-sectional study collecting data between June 2017 and March 2018in the pre-operative Eye Clinic of Hospital Melaka. Analysis was performed after data collection via the use of descriptive and inferential statistics. National Eye Institute Visual Functioning Questionnaire − 25 (VFQ-25) version 2000 is an interviewer administered format available online which allows measurement of important health status of people with chronic eye diseases [7].

The original version of the VFQ-25 Questionnaire was in English but with majority of our population being able to converse and understand the Malay language which is our National language, we translated the English version into the Bahasa Malaysia version to cover population who are not literate in English.

The study was done to compare the validation properties of the English version of the Questionnaire and the Bahasa Malaysia (Malay Language) version regarding the Quality of Life of patients with cataracts. The study period included writing the proposal, collecting and analysing the data and completing the write-up. This VFQ-25 questionnaire was utilized to assess the quality of life in terms of problems of vision and feelings about vision among cataract patients in Melaka, Malaysia. The English version of the Questionnaire was obtained from National Eye Institute Visual Functioning Questionnaire − 25 (VFQ-25) version 2000.

We conducted the test on 428 participants who had cataract in the pre-operative Eye Clinic of Hospital Melaka. The English version of the Questionnaires were handed out to 224 patients while Questionnaires in Malay Language were given to 204 participants.

Forward Translation: The translation of the VFQ-25 Questionnaire was performed separately by two independent translators who was conversant in both Bahasa Malaysia and the English language. We instructed the translators to use common Bahasa Malaysia equivalents for phrases and words and to translate the questionnaire as carefully and closely as possible.

Backward Translation: The backward translation of the Bahasa Malaysia version of the VFQ-25 Questionnaire to English was performed by a different translator who was well versed in both languages. Assessment of the backward translation with the original English version was made by a group made up of the researchers and an independent professional. The assessment centred on the conceptual equivalence of both Backward Translation version with the original version. 94 % of items were noted to be conceptually equivalent and the final Bahasa Malaysia version of the VFQ-25 Questionnaire approved.

The patients were given consent form to sign to participate in the study. The Questionnaire was distributed by independent persons who were not part of the study. These data enumerators were trained on data collection methods.

Methods used to validate the standard questionnaire included the use of construct validity via factor analysis and the deployment of reliability test through assessment of internal consistency via Cronbach’s alpha. We regarded Cronbach’s alpha values of 0.65 and above as fair and adequate for reliability testing.

The initial draft of the VFQ-25 contained 3 main parts and 8 subscales which included looking into the following domains - difficulty in performing activity in daily living, mental outlook/perception and personal satisfaction.

A small pilot study was performed on 10 participants each for both the English and Bahasa Malaysia versions of the questionnaire. 90 % indicated that they understood the questions and found them easy to answer in both the English and Bahasa Malaysia versions respectively.

### Statistical analysis

For construct validity and reliability tests, we analyzed our data by using SPSS Version 25. We began statistical analysis by examining our data using descriptive statistics which looks into the mean and standard deviation of each item in both English and Bahasa Malaysia versions of the questionnaire.

### Construct validity

For construct validity, we utilized factor analysis which is a statistical instrument used to condense and consolidate the items within the questionnaire into factors. These items are loaded into common factors with the main aim of consolidating the items into a small number of factors [10]. Loading here denotes the measure of association between an item and a factor.

We performed test to examine the adequacy of the sample and the suitability of data for factor analysis by examining the Kaiser-Meyer Olkin [11, 12].

For data to be of value to our research, we utilized the following statistical cut-off values and ranges - correlation coefficient values of 0.3 to 0.9, P value of correlation coefficient at less than 0.05 and Kayser Myer Olkin (KMO) sampling adequacy above 0.5 [13].

### Rotation

We employed rotation to simplify and clarify the data structure. Rotation ensures maximum loading of each variable (item) into one extracted factor while at the same time ensuring that this same variable is not loaded into the other factors. We utilized orthogonal; methods such as Varimax, Quartimax and Equamax when variables were assumed to be orthogonal (independent of each other). For variables that were dependent of each other (oblique), we considered the use of Direct Oblimin and Promax. The usage of Equamax in our statistical study allowed us to produce factors that are uncorrelated.

### Comparison

We performed factor analysis on both the English and Bahasa Malaysia versions of the VFQ-25 Questionnaire separately. We identified items produced under each component (factor) in both versions and we then proceeded to compare the English version to the Bahasa Malaysia version with regards to the number of components and items within these components.

### Item Response Theory (IRT)

We proceeded to conduct item response theory which allows assessment of participants’ responses to individual items within the VFQ-25 Questionnaire so as to assist in identifying the quality of those items and of the questionnaire as a whole. We utilized R studio incorporating R version 3.6.3 for the conduct of the IRT analyses. Generalized Partial Credit Model (GPCM) of the item response theory was utilized to suit the polytomous ordinal nature of responses required by the questionnaire [14]. Each of the components (factors) that were produced via factor analysis was analyzed separately so as enable analysis of each item’s parameters which include item discrimination, item difficulty and item fit.

Each item in the questionnaire was assessed for its quality in fitting into the components (factors) based on two parameters - the root mean square error of approximation (RMSEA) and the P value for the Chi-Square for Goodness of Fit. For the RMSEA, anything less than < 0.06 is considered to reflect good fit while values < 0.08 are considered fair and finally values which are above 0.10 are generally considered as poor fit [15]. The *P* value for the Chi-Square for Goodness of Fit should also be more than 0.05 to signify good fit. The RMSEA is considered first in decision making whenever there are differences between the two (RMSEA and Chi-square).

We analyzed each item for its discrimination properties with values of more than 0.4 as good, 0.2 to 0.4 as acceptable and anything less than 0.2 as poor [16].

### Ethical consideration

The approval to conduct this research was granted by the Medical Review and Ethics Committee (MREC) Ministry of Health Malaysia (06/02/2017). All participants gave written consent to participate in the study. This form was checked and approved by MREC.

## Results

A total of 224 respondents took part in the analysis of the English version of the National Eye Institute Visual Functioning Questionnaire while another 204 participated in the Malay version.

The mean age of these patients was 66.8 years of age and 64.3 years of age for those involved in the English version and Bahasa Malaysia version respectively (Not In Table). The respondents were made up of 52.5 % males in the English version and 49.8 % males in the Bahasa Malaysia version. The main ethnic groups in the English version database comprised of Malays (56.1 %) followed by Chinese (29.8 %) and the Indians (14.1 %) while the Bahasa Malaysia version database consisted of Malays (53.7 %) followed by Chinese (31.2 %) and the Indians (15.1 %).

All 25 items from the English version and Bahasa Malaysia version of the questionnaires were analyzed for the component factor analysis, of which 19 items each were loaded into the English and Bahasa Malaysia versions respectively. Six items each from the English and Bahasa Malaysia version of the Questionnaire were suppressed from further analysis as they had loadings of less than 0.4.

As illustrated in Table 1, Kaiser-Meyer-Olkin’s measure of sampling adequacy indicated excellent compactness with regards to the pattern of correlations with values of 0.923 for the English questionnaire and 0.927 for the Bahasa Malaysia questionnaire (P value < 0.05 for Bartlett’s Test). These results allowed us to proceed with factor analysis.

**Table 1:** Kaiser-Meyer-Olkin Measure of Sampling Adequacy of the English and Bahasa Malaysia Questionnaire in assessing problems and perceptions of vision, Hospital Melaka, 2018

Test	English Questionnaire	Bahasa Malaysia Questionnaire
Kaiser-Meyer-Olkin Measure of Sampling Adequacy	0.923	0.927
Bartlett’s Test of Sphericity	*P* < 0.001***	*P* < 0.001***

*** *P* value < 0.05 Significant

We performed extraction of components followed by the use of all rotation types possible. Bearing in mind that items are independent of each other, we utilized Varimax rotation followed by Equamax rotation and finally ending with Quartimax rotation. We finally settled for the use of Equamax rotation as this rotation produces the least discrepancies in percentage of variance among the various components produced with Eigenvalue of more than one.

As shown in Table 2, a total of three components have eigenvalues of more than one with cumulative percentage of 60.7 % for the English version of the questionnaire. Components one, two and three contributed 23.8 %, 22.2 and 14.6 % towards the total variance, respectively. As for the Bahasa Malaysia version of the questionnaire, similarly a total of three components presented with eigenvalues of one and above (Table 2). These three components accounted 24.2 %, 23.6 and 18.6 % respectively towards the total variance and together contributed a cumulative variance of 66.5 % which was approximately 5.8 % higher that the cumulative variance seen in the English version of the questionnaire.

**Table 2:** Total variance explained –Initial Eigenvalues and rotation sum via Equamax Rotation of the English and Bahasa Malaysia Questionnaire in assessing problems and perceptions of vision, Hospital Melaka, 2018

		Initial Eigenvalues			Rotation sum of squared loadings (Equamax Rotation)		
Components		Total	% of Variance	Cumulative %	Total	% of Variance	Cumulative %
**English Questionnaire**							
Component 1		8.42	44.34	44.34	4.52	23.81	23.81
Component 2		1.77	9.35	53.70	4.22	22.22	46.04
Component 3		1.33	7.01	60.72	2.79	14.68	60.72
**Bahasa Malaysia Questionnaire**							
Component 1		9.82	51.72	51.72	4.61	24.28	24.28
Component 2		1.82	9.58	61.31	4.49	23.66	47.94
Component 3		1.00	5.25	66.56	3.53	18.62	66.56

In the English version of the questionnaire, nine items loaded strongly onto component one, six items were loaded onto component two and a further three items onto component three (Table 3). As for the Bahasa Malaysia version of the questionnaire, we observed loadings of 10 items, seven items and one item respectively onto components one, two and three respectively (Table 4).

**Table 3:** The results of the final three factor solution of the English Questionnaire in assessing problems and perceptions of vision, via the use of Principal Component Analysis with Equamax rotation, Hospital Melaka, 2018

Items	Factor 1 Loadings	Factor 2 Loadings		Factor 3 Loadings
**Difficulty with activities**				
B2Q5. How much difficulty do you have reading ordinary print in newspapers?	0.639			
B2Q6. How much difficulty do you have doing work or hobbies that require you to see well up close, such as cooking, sewing, fixing things	0.673			
B2Q8.How much difficulty do you have reading street signs or the names of stores?	0.699			
B2Q9. Because of your eyesight, how much difficulty do you have going down steps, stairs, or curbs in dim light or at night?	0.674			
B2Q10. Because of your eyesight, how much difficulty do you have noticing objects off to the side while you are walking along?	0.737			
B2Q11. Because of your eyesight, how much difficulty do you have seeing how people react to things you say?	0.702			
B2Q12. Because of your eyesight, how much difficulty do you have picking out and matching your own clothes?	0.620			
B2Q13. Because of your eyesight, how much difficulty do you have visiting with people in their homes, at parties, or in restaurants?	0.673			
B2Q14. Because of your eyesight, how much difficulty do you have going out to see movies, plays, or sports events?	0.597			
**Responses to vision problem**				
B3Q20. I stay home most of the time because of my eyesight.		0.714		
B3Q21. I feel frustrated a lot of the time because of my eyesight.		0.695		
B3Q22.I have much less control over what I do, because of my eyesight.		0.722		
B3Q23. Because of my eyesight, I have to rely too much on what other people tell me.		0.778		
B3Q24. I need a lot of help from others because of my eyesight.		0.748		
B3Q25. I worry about doing things that will embarrass myself or others, because of my eyesight.		0.760		
**General health and vision**				
B1Q2.At the present time, would you say your eyesight using both eyes (with glasses or contact lenses, if you wear them) is excellent, good, fair, poor, or very poor or are you completely blind?				0.601
B1Q3. How much of the time do you worry about your eyesight?				0.578
B2Q5. How much difficulty do you have reading ordinary print in newspapers?				0.422

**Table 4:** The results of the final three factor solution of the Bahasa Malaysia Questionnaire (presented here in English) in assessing problems and perceptions of vision, via the use of Principal Component Analysis with Equamax rotation, Hospital Melaka, 2018

Items	Factor 1 Loadings	Factor 2 Loadings		Factor 3 Loadings
**Difficulty with activities**				
B1Q2.At the present time, would you say your eyesight using both eyes (with glasses or contact lenses, if you wear them) is excellent, good, fair, poor, or very poor or are you completely blind?	0.678			
B2Q5. How much difficulty do you have reading ordinary print in newspapers?	0.786			
B2Q6. How much difficulty do you have doing work or hobbies that require you to see well up close, such as cooking, sewing, fixing things	0.557			
B2Q8.How much difficulty do you have reading street signs or the names of stores?	0.766			
B2Q9. Because of your eyesight, how much difficulty do you have going down steps, stairs, or curbs in dim light or at night?	0.718			
B2Q10. Because of your eyesight, how much difficulty do you have noticing objects off to the side while you are walking along?	0.561			
B2Q11. Because of your eyesight, how much difficulty do you have seeing how people react to things you say?	0.555			
B2Q12. Because of your eyesight, how much difficulty do you have picking out and matching your own clothes?	0.479			
B2Q13. Because of your eyesight, how much difficulty do you have visiting with people in their homes, at parties, or in restaurants?	0.526			
B2Q14. Because of your eyesight, how much difficulty do you have going out to see movies, plays, or sports events?	0.641			
**Responses to vision problem**				
B3Q20. I stay home most of the time because of my eyesight.		0.707		
B3Q21. I feel frustrated a lot of the time because of my eyesight.		0.731		
B3Q22.I have much less control over what I do, because of my eyesight.		0.766		
B3Q23. Because of my eyesight, I have to rely too much on what other people tell me.		0.833		
B3Q24. I need a lot of help from others because of my eyesight.		0.751		
B3Q25. I worry about doing things that will embarrass myself or others, because of my eyesight.		0.793		
B3Q18 Are you limited in how long you can work or do.		0.490		
**Less accomplishment due to poor eyesight**				
B3Q17.Do you accomplish less than you would like because of your vision?				-0.777

The loading process in component one (defined here as “Difficulty in Activity of Daily Living”) revealed nine similar items for both English and Bahasa Malaysia versions of the questionnaire (Tables 3 and 4).

As for component two (defined here as “Dependency on others due to poor eyesight”), the loading revealed six similar items for both English and Bahasa Malaysia versions of the questionnaire (Tables 3 and 4).

The English version of the National Eye Institute Visual Functioning Questionnaire comprises of three components:

1) Component one: “Difficulty with activities”, which accounted for 23.8 % of the total variance. This component contained nine items and reflected perception of the difficulty in activity of daily living. The highest loading items were as follows – “Because of your eyesight, how much difficulty do you have noticing objects off to the side while you are walking along?” (factor loading of 0.73); “Because of your eyesight, how much difficulty do you have seeing how people react to things you say?” (factor loading of 0.84); “How much difficulty do you have reading street signs or the names of stores?” (factor loading of 0.69).

2) Component two: “Responses to vision problems”, which comprised of 22.2 % of the total variance. Six items were included within this component which reflected perception on dependency on others due to poor eyesight. We noted the highest loading items as follows – “Because of my eyesight, I have to rely too much on what other people tell me.“ (factor loading of 0.77); “I worry about doing things that will embarrass myself or others, because of my eyesight.“ (factor loading of 0.76); “I need a lot of help from others because of my eyesight.“ (factor loading of 0.74).

3) Component three: “General health and vision”, which contributed 14.6 % of the total variance. This component comprised of three items which portrayed poor perception of respondents on status of eyesight. The following items were loaded highest as follows –.

“At the present time, would you say your eyesight using both eyes (with glasses or contact lenses, if you wear them) is excellent, good, fair, poor, or very poor or are you completely blind?“ (factor loading of 0.60); “How much of the time do you worry about your eyesight?“ (factor loading of 0.57); “How much difficulty do you have reading ordinary print in newspapers?“ (factor loading of 0.42).

The final Bahasa Malaysia version of the National Eye Institute Visual Functioning Questionnaire constitutes the following three components:

1) Component one: “Difficulty with activities”, contributing towards 23.6 % of the total variance. A total of ten items were loaded which reflected perception of the difficulty in activity of daily living. The highest loading items were as follows – “How much difficulty do you have reading ordinary print in newspapers?“ (factor loading of 0.78); “How much difficulty do you have reading street signs or the names of stores?“ (factor loading of 0.76); “Because of your eyesight, how much difficulty do you have going down steps, stairs, or curbs in dim light or at night?“ (factor loading of 0.71).

2) Component two: “Responses to vision problems”, comprising of 24.2 % of the total variance. We observed seven items that were included within this component which reflected perception on dependency on others due to poor eyesight. The highest loading items were as follows –.

“Because of my eyesight, I have to rely too much on what other people tell me.“ (factor loading of 0.83); “I have much less control over what I do, because of my eyesight.“ (factor loading of 0.76); “I feel frustrated a lot of the time because of my eyesight.“ (factor loading of 0.73).

3) Component three: “Poor perception of status of eyesight”, accounting for 18.6 % of the total variance. There was only one item loaded into the component which portrayed poor perception of respondents on status of eyesight. The lone item loaded was noted as follows –.

“Do you accomplish less than you would like because of your vision?“ (factor loading of -0.77).

### Reliability – internal consistency

After construct validation was computed, Cronbach’s alpha was computed for the revised questionnaire and we obtained Cronbach alpha values of 0.904, 0.898 and 0.608 for the three components of the English version of the questionnaire – difficulty with activities, responses to vision problems and general health and vision. As for the Bahasa Malaysia version, computation of Cronbach’s alpha revealed values of 0.921, 0.927 for the first two components - difficulty with activities, responses to vision problems. Cronbach’s alpha was not done on Component 3 (general health and vision) of the Bahasa Malaysia version as Component 3 has only one item.

### Item response theory

We then proceeded to perform the Item Response Theory analysis via the use of the GPCM model. We analyzed separately each of the components (factors) that were produced via factor analysis earlier for both the English and Bahasa Malaysia questionnaires.

Table 5 illustrates the parameter estimates and fit statistics for the English questionnaire. All three components indicated good to excellent fit of the items towards each of the components with the root mean square error of approximation (RMSEA) values of less than 0.06 and *P* value (for the Chi-square Goodness of Fit Test) > 0.05 for all items. In addition, the items displayed high discrimination with values ranging from 0.74 to 1.79 for Component one: “Difficulty with activities”; values ranging from 0.90 to 2.08 for Component two: “Responses to vision problems”; and 0.65 to 1.25 for Component three: “General health and vision”.

**Table 5:** GPCM item parameters’ estimates and fit statistics performed separately for each of the three-factor solution of the English Questionnaire, Hospital Melaka, 2018

Item	a	b1	b2	b3	b4	b5	Chi-Square	df	RMSEA	*P* value
**Difficulty with activities**										
B2Q5	0.74	-2.20	-0.67	1.70	0.53	2.99	43.16	41	0.016	0.379
B2Q6	1.28	-1.65	-0.37	1.21	1.85	2.03	45.35	34	0.039	0.092
B2Q8	1.55	-1.26	-0.38	0.72	2.01	2.63	29.41	31	0.000	0.548
B2Q9	1.72	-1.49	-0.47	0.60	1.80	2.67	36.80	29	0.035	0.151
B2Q10	1.79	-1.23	-0.25	0.66	2.32		33.51	29	0.027	0.258
B2Q11	1.70	-0.69	-0.22	1.42	2.17		25.62	30	0.000	0.694
B2Q12	1.45	0.04	0.43	1.75	2.54	2.53	20.95	30	0.000	0.889
B2Q13	1.68	-0.39	0.48	1.47	2.13	2.28	36.09	31	0.027	0.243
B2Q14	0.78	-0.90	0.05	1.18	1.03	1.39	67.02	51	0.038	0.066
**Responses to vision problem**										
B3Q20	1.66	-1.56	-0.05	0.06	0.67		32.08	30	0.018	0.364
B3Q21	1.62	-1.29	0.04	0.24	1.22		31.74	34	0.000	0.579
B3Q22	1.72	-1.54	-0.16	0.42	1.28		32.46	30	0.019	0.346
B3Q23	2.08	-1.84	-0.39	0.04	0.79	3.37	30.11	23	0.037	0.146
B3Q24	1.91	-3.44	-1.93	-0.15	-0.17	0.98	31.83	24	0.038	0.131
B3Q25	0.90	-2.45	-0.47	-0.18	0.33	4.60	41.23	31	0.039	0.104
**General health and vision**										
B1Q2	1.25	-3.75	-1.15	0.17	2.17		7.28	7	0.013	0.400
B1Q3	0.65	-3.11	-1.97	-0.11	1.77		11.25	10	0.024	0.339
B2Q5	0.70	-2.24	-0.72	1.73	0.57	3.19	4.57	6	0.000	0.600

Note: a refers to item discrimination; b1 to b5 are values for item difficulty and obtained as result of polytomous nature of Likert scale – an item with a Likert scale of 6 would have values till b5, Likert scale of 5 till b4; RMSEA stands for root mean square error of approximation; Chi-Square calculated for Goodness of Fit with subsequent df (degree of freedom) and *P* value

**Fig. 1:**
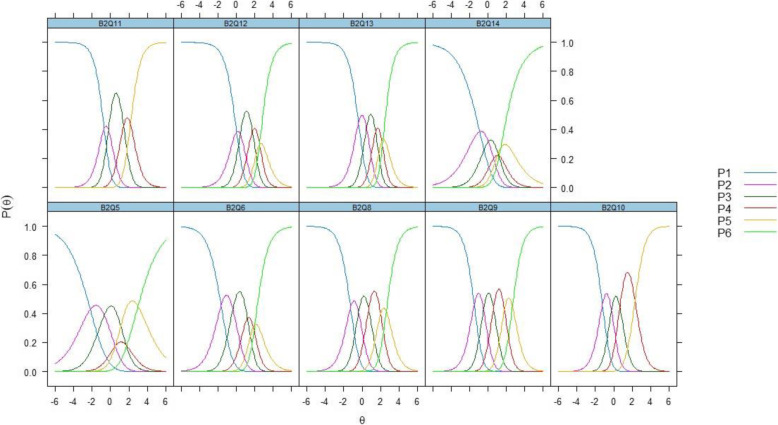
Item characteristic curves for items under Component 1:”Difficulty with activities” with P(θ) representing proportion/probability correct and θ overall measure of ability of respondents

Figure 1 displays the item characteristic curves (ICC) with curves comprising of P1 and up to P5 or P6 representing the Likert scales utilized in each item – e.g. Likert scale 1 for P1 curve, Likert scale 2 for P2 curve and so on. Those scoring P1 (lowest scale) were more likely seen to the left of each ICC indicating low scoring amongst those with low ability (low “difficulty with activities”) while conversely those scoring P5 or P6 (highest scale) were more likely seen towards the right of the ICCs pointing to high scoring amongst those with high ability (high “difficulty with activities”). Visually, the curves for all of the items indicated that they are well constructed items as there are adequate ordering of these curves within each item from the lowest scale on the left to the highest scale on the right. In addition, the items between the extremes (P2 curve to either P4 or P5) are bunched right in between the first (P1) and the last scale curve (P5 or P6). For instance, a poorly constructed item would have their peaks either to the left of the P1 curve or to the right of the last curve (P5 or P6).

As per Table 6, similar results were seen in the Bahasa Malaysia questionnaire with most items displaying good to excellent fit (RMSEA values of less than 0.06) towards the two analyzed components - Component one: “Difficulty with activities”, Component two: “Responses to vision problems”. Three items (B2Q12, B2Q13, B2Q14) in Component one: “Difficulty with activities” and a further three items (B3Q21, B3Q24, B3Q18) in Component two: “Responses to vision problems” have RMSEA values between 0.06 and 0.08 and thus considered to be fair fit towards their respective components (factors). Moreover, the items illustrated high discrimination with values ranging from 0.78 to 2.29 for Component one: “Difficulty with activities” and values ranging from 1.08 to 4.35 for Component two: “Responses to vision problems”.

**Table 6:** GPCM item parameters’ estimates and fit statistics performed separately for each of the three-factor solution of the Bahasa Malaysia Questionnaire, Hospital Melaka, 2018

Item	a	b1	b2	b3	b4	b5	Chi-Square	df	RMSEA	*P* value
**Difficulty with activities**										
B1Q2	0.78	-4.65	-0.73	0.34	2.32		60.66	36	0.060	0.006
B2Q5	1.27	-1.95	-0.54	0.56	1.12	1.98	57.47	37	0.054	0.017
B2Q6	1.09	-1.89	-0.60	0.95	1.44	2.82	50.71	33	0.053	0.025
B2Q8	2.04	-1.26	-0.29	0.49	1.27	2.55	50.12	30	0.060	0.012
B2Q9	2.00	-1.64	-0.31	0.54	1.54	1.53	39.97	26	0.053	0.039
B2Q10	2.29	-1.15	-0.23	0.97	1.53	2.31	36.27	26	0.046	0.087
B2Q11	2.10	-0.90	-0.15	1.09	1.85	2.57	45.44	27	0.060	0.015
B2Q12	1.35	-0.34	-0.03	1.81	1.72	1.74	52.19	28	0.068	0.004
B2Q13	2.18	-0.72	0.08	1.11	1.38	2.75	56.11	27	0.076	0.001
B2Q14	0.98	-0.65	-0.39	1.51	0.11	1.07	88.46	42	0.077	0.000
**Responses to vision problem**										
B3Q20	1.82	-1.49	0.27	-0.39	1.62		38.61	24	0.057	0.030
B3Q21	1.74	-1.76	0.42	-0.24	1.52	3.45	46.66	22	0.077	0.002
B3Q22	2.50	-1.67	0.08	0.35	1.27		12.07	20	0.000	0.913
B3Q23	4.35	-1.59	-0.16	-0.05	1.11	2.76	15.31	15	0.011	0.429
B3Q24	2.42	-1.49	0.27	-0.23	1.30	3.13	33.02	18	0.067	0.017
B3Q25	2.17	-1.82	-0.01	-0.37	1.18		28.76	23	0.037	0.188
B3Q18	1.08	-0.81	-0.37	-0.26	1.40		73.51	34	0.079	0.000
**Less accomplishment due to poor eyesight**										
B2Q5	Insufficient degree of freedom as there is only one item									

Note: a refers to item discrimination; b1 to b5 are values for item difficulty and obtained as result of polytomous nature of Likert scale – an item with a Likert scale of 6 would have values till b5, Likert scale of 5 till b4; RMSEA stands for root mean square error of approximation; Chi-Square calculated for Goodness of Fit with subsequent df (degree of freedom) and P value

## Discussion

The National Eye Institute Visual Functioning Questionnaire – 25 (NEI VFQ 25) has been designed to measure important areas of well-being and functioning among patients with eye diseases [17]. It has been further described as a valid and reliable tool in assessing vision-specific quality of life among patients with various eye diseases [18].

The ultimate aim of this study was to compare the psychometric properties of the English version of the Questionnaire and the Bahasa Malaysia (Malay Language) version regarding the vision-related Quality of Life of patients with cataracts. We assessed the reliability and validity of the English and Bahasa Malaysia version of the NEI VFQ-25 in ophthalmic patients with cataract diseases.

The original National Eye Institute Visual Functioning Questionnaire – 25 which was first developed by RAND [19] is comprised of three factors which touched upon general health and vision, difficulty with activities and responses to vision problem.

We observed both English and Bahasa Malaysia versions to have high reliability with Cronbach’s alpha values of 0.90 and above in factors on difficulty with activities and responses to vision problems which mirrored results seen in studies validating the questionnaire in Japanese [20], Italian [21], French [22], Spanish [23], Turkish [24] and German [25].

With regards to construct validity, exploratory factor analysis performed has revealed that the three-factor model fits the data well for the English version of the questionnaire. Extraction and rotation of the data produced a model that mirrored the original version with regards to production of a total of three factors – difficulty with activities (23.81 % of variance), responses to vision problem (22.22 % of variance) and general health and vision (14.68 % of variance). The items were mostly similar between the model and the original questionnaire in these three factors– there were a total of nine identical items shared in the factor on difficulty with activities, six identical items shared in the factor on responses to vision problem and two similar items shared in the factor on general health and vision. The results supported a three-factor solution as per original version of the questionnaire, with the factors accounting for 60.72 % of the variance.

However, there were a couple of items left out in the model. For instance, on difficulty with activities, two items which pertain to driving at night and driving in difficult conditions were omitted from the model. Similarly, a study by Lloyd et al. noted that driving in difficult conditions or at night items merged out into a totally separate domain [26].

A further three items on accomplishment, limitation of work activities and pain discomfort were left out from the model in the factor on responses to vision problems.

As similarly seen in the English version, the Bahasa Malaysia version of the questionnaire produced three factors with two of the factors resembling the factors from the original version of the questionnaire - difficulty with activities (24.28 % of variance) and responses to vision problem (23.66 % of variance). In addition, there was higher cumulative variance in the Bahasa Malaysia version (66.56 % Bahasa Malaysia version versus 60.72 % English version) indicating stronger fit to the model for the Bahasa Malaysia version as compared to the English version. Altogether, there were a total of ten identical items shared in the factor on difficulty with activities and seven identical items shared in the factor on responses to vision problem between the Bahasa Malaysia and the original version of the questionnaire.

Factor analysis on the Brazilian version of the NEI VFQ-25 indicated that the tool could be utilized in the country to assess vision-related quality of life as the psychometrics properties are comparable to the original American version [27]. The Japanese version of the questionnaire also noted similar observation [20].

However, in comparison to these studies conducted in Brazil [27] and Japan [20] which focused on subscales derived from the optional questions rather than the main parts of the questionnaire, our research revealed three factor model – difficulty with activities, responses to vision problem and general health and vision which mirrored precisely with the main parts of the original NEI VFQ-25 questionnaire (Part 2, Part 3 and Part 1 respectively). In addition, as the population of Malaysia are well conversant in both the Malay and English languages, we were able to perform validation analyses for questionnaires in both languages and ultimately witness the high reliability and validity in these questionnaires in assessing the quality of life of people with cataract.

With good reliability, validity and good to excellent fit of items within the questionnaire, the VFQ-25 has been utilized in surveys involving ophthalmic patients with diabetic retinopathy, primary open-angle glaucoma, cataract, low vision, optic neuritis, uveitis and others such as age-related macular degeneration and cytomegalovirus retinitis [9, 28–31].

This study with good psychometric features could be used as a routine tool to assess visual function among patients especially among rural folks who predominantly understand Bahasa Malaysia which is the official language in Malaysia. In addition, this study validates the use of the English version of the questionnaire which may be utilized by those who prefer the use of the English language which is especially seen among the urban population of Malaysia.

## Conclusions

The present study observed that both the English and Bahasa Malaysia versions of the NEI VFQ-25 have comparable construct validity to the original American version. With high validity and reliability, the tool shall be able to provide health care providers the assessment of impact due to cataract and other ophthalmic conditions on the vision-related quality of life of ophthalmic patients.

## Data Availability

All data is contained within the manuscript and its supplementary files.

## Abbreviations

NEI VFQ: National Eye Institute Visual Functioning Questionnaire
MREC: Medical Research Ethics Committee
